# Targetable alterations in primary extranodal diffuse large B‐cell lymphoma

**DOI:** 10.1002/jha2.428

**Published:** 2022-05-23

**Authors:** Stephanie E. Weissinger, Rucha Dugge, Miriam Disch, Thomas F. Barth, Johannes Bloehdorn, Malena Zahn, Ralf Marienfeld, Andreas Viardot, Peter Möller

**Affiliations:** ^1^ Institute of Pathology University Hospital Ulm Ulm Germany; ^2^ Institute of Pathology Alb Fils Kliniken GmbH Göppingen Germany; ^3^ Department of Internal Medicine III University Hospital Ulm Ulm Germany

**Keywords:** extranodal diffuse large B‐cell lymphoma, immunohistochemical analysis, mutational analysis, prognosis, targeted therapy

## Abstract

Primary extranodal diffuse large B‐cell lymphoma (PE‐DLBCL) is a heterogeneous subgroup of DLBCL. We investigated the prevalence and prognostic value of surface expression of PD‐L1, PD1, and CD30, copy number of 9p24.1 (PD‐L1 region), and mutations in *MYD88*, *CD79B*, *CARD11*, and *BTK* in a cohort of 116 patients, localized in the mediastinum (PMBL, *n* = 12), ear, nose and throat (ENT, *n* = 28), central nervous system (*n* = 29), testis (*n* = 7), breast (*n* = 4), stomach (*n* = 10), bone (*n* = 8), spleen (*n* = 2), and skin (*n* = 16). PD‐L1 expression is most frequent in PMBL (92%), followed by lymphomas originating in the stomach (57%), ENT (23%), and skin (18%). PD1 was expressed at low levels in less than 13% of PE‐DLBCL, while CD30 expression was found in 58% of PMBL. Mutation analysis revealed an unexpectedly high frequency of *MYD88* and *CD79B* mutations in ENT lymphomas (46% and 50%, respectively). *CARD11* mutations are rare but more frequently found in gastric lymphomas (30%), suggesting BTK resistance. Thirty‐four of 113 (30%) of the lymphomas harbored both *MYD88* and *CD79B* mutations. Lower overall and progression‐free survival rates were found for cases with *MYD88*, *CD79B*, and *BTK* mutations. These data confirm the biologic singularity of PE‐DLBCLs and provide some suggestions for targeted therapies.

## 1 INTRODUCTION

Approximately 25%–40% of non‐Hodgkin lymphomas (NHLs) have extranodal manifestations [1, 2]. Patients with clearly defined primary extranodal diffuse large B‐cell lymphomas (PE‐DLBCLs) are less frequent. They represent a heterogeneous group of NHLs with pronounced differences in clinical manifestation, treatment approach, and particularly in outcome.

Some aberrations are more frequent in extranodal lymphomas, including attribution to the “ABC” (activated B‐cell) or non germinal center B‐cell (non‐GCB) type by gene expression profiling [3], a high frequency of *MYD88* and *CD79B* mutations [4, 5, 6], PD‐L1 expression linked to genomic changes at the chromosomal region 9p24.1 [7], CD30 [8] expression, and positivity for the Epstein–Barr virus (EBV) [9]. In the light of the novel molecular classifications of DLBCL using the Chapuy [5] and Schmitz [4] models, two molecular patterns are enriched particularly in PE‐DLBCLs: the “cluster 5” or “MCD‐type” based on *MYD88* and *CD79B* mutations and the “cluster 2” or “BN‐2” type, based on *BCL6* fusions and *NOTCH2* mutations, which are associated with MALT lymphomas. From a clinical viewpoint, the MCD‐type might be highly responsive to inhibition of BTK [10]. Expression of PD‐L1, partially with copy gains in 9p24.1, is a hallmark of primary mediastinal B‐cell lymphoma [11] and explains the lymphomas of the central nervous system (CNS) and testis [12], but data from other entities are scarce. CD30 expression is rare in DLBCL (approximately 15%) but might be enriched in lymphoma with extranodal involvement [8]. CD30 is a target for anti‐CD30 antibody drug conjugates such as brentuximab vedotin [13]. While the patterns of these targetable alterations are described comprehensively in a few subentities (e.g., CNS lymphoma [12, 14] and PMBL [15]), data from other subentities are limited. Therefore, we analyzed a large cohort of homogeneously treated (chemoimmunotherapy) patients with PE‐DLBCLs (stage I, stage II with small locoregional lymph nodes, and, by definition, PMBL) for the prevalence, patterns, and prognostic significance of these targetable aberrations.

## 2 METHODS

The cohort consisted of 116 patients, diagnosed with and treated for PE‐DLBCL at the Ulm University Hospital, Germany, between 2002 and 2018. PE‐DLBCL was defined as distinctive PE involvement based on biopsies taken from an extranodal site. PE‐DLBCL of all localizations diagnosed during this period was included, regardless of the number of cases of the respective localization. Stage II patients were not included unless only small locoregional lymph nodes were involved. Patients were staged according to the Ann Arbor classification and the International Prognostic Index (IPI) [16, 17, 18]. Patients with systemic disease or primary nodal DLBCL were excluded from the study. Lymphoma typing was done according to World Health Organization (WHO) guidelines [19]. Treatment regimens were R‐CHOP (Rituximab, Cyclophosphamide, Doxorubicin, Vincristine, Prednisone) (45/95, 47%) or intensified treatment regimens such as R‐CHOEP and R‐DHAP (4/95; 4%), R‐bendamustine (5/95; 5%) or others (41/95; 43%, with mainly CNS lymphomas being treated with R‐methotrexate containing regimens). In total, 88/95 (93%) of the patients were treated by immunochemotherapy. Seven patients received radiotherapy or active surveillance due to advanced age. All formalin fixed and paraffin embedded (FFPE) tissues examined were from biopsies or specimen excisions prior to therapy. The study was approved by the local ethics committee (application number: 381/17).

For determining the subtype antibodies against CD20, CD10, Mum1, and Bcl6 were used. PD1, PD‐L1, and CD30 were used for biomarkers. Gene copy number variations of the *PDL1/2* gene on chromosome 9p24.1 were assessed using a *CD274/CEN9* dual color fluorescence in situ hybridization (FISH) probe. Samples were sequenced for *MYD88, CD79B, CARD11*, and *BTK* genes. Details, as well as methods for *generating heatmap, Kaplan–Meier curves*, and *statistical analysis* are provided in the supplement.

## 3 RESULTS

### 3.1 Cohort characteristics

The cohort consisted of 116 patients: 49 (42%) females and 67 (58%) males. Extranodal DLBCL involved the mediastinum (PMBL, *n* = 12), ear, nose, and throat (ENT) (*n* = 28), CNS (*n* = 29), testis (*n* = 7), breast (*n* = 4), stomach (*n* = 10), bone (*n* = 8), spleen (*n* = 2), and skin (*n* = 16). Median age of the patients was 66 years. The cohort comprised patients with Ann Arbor stage I (*n* = 79; 73%), stage II (*n* = 26; 24%), and stage IV (*n* = 3; 3%). All three stage IV patients had multifocal skin involvement. No stage III patients were included (Table 1).

**TABLE 1:** Study cohort characteristics and results

Characteristics	Total *N* = 116	PMBL *N* = 12	ENT *N* = 28	CNS *N* = 29	Testis *N* = 7	Breast *N* = 4	Gastric *N* = 10	Skin *N* = 16	Bone *N* = 8	Spleen *N* = 2
**Sex, *n* (%)**										
Male	67/116(58)	8(67)	13(46)	14(48)	7(100)	–	4(40)	13(81)	5(62)	1(50)
Female	49/116(42)	4(33)	15(53)	15(52)	–	4(100)	6(60)	3(19)	3(37)	1(50)
**Age in years**										
Median at PD	66	41	68	65	66	65	71	70	50	62
**Stage (%)**										
I	79/108(73)	–	20/28(71)	29/29(100)	3/5(60)	4(100)	8(80)	8/12(67)	5/7(71)	2(100)
II	26/108(24)	12/12(100)	8/28(28)	–	2/5(40)	–	2(20)	1/12(8)	2/7(29)	–
III	–	–	–	–	–	–	–	–	–	–
IV	3/108(3)	–	–	–	–	–	–	3/12(25)	–	–
**IPI**										
0	17/84(20)	1/12(8)	7/27(26)	3/27(11)	–	2/4(50)	–	1/7(14)	3/7(43)	–
1	42/98(43)	7/12(60)	13/27(50)	8/27(30)	2/5(40)	2/4(50)	5/9(56)	3/7(43)	2/7(29)	–
2	31/94(33)	4/12(33)	6/27(22)	12/27(44)	3/5(60)	–	2/9(22)	3/7(43)	1/7(14)	–
3	8/90(9)	–	1/27(4)	4/27(15)	–	–	2/9(22)	–	1/7(14)	–
**Subtype**										
GCB	27/99(27)	–	8/28(28)	6/29(21)	1/7(14)	0/4(0)	2/7(28)	4/16(25)	5/7(71)	1/2(50)
non‐GCB	73/99(74)	–	20/28(71)	23/29(79)	6/7(86)	4/4(100)	5/7(71)	12/16(75)	2/7(28)	1/2(50)
**EBV**										
Positive	3/109(3)	2/11(18)	0/28(0)	1/29(3)	0/6(0)	0/4(0)	0/8(0)	0/15(0)	0/7(0)	0/2(0)
Negative	106/109(97)	9/11(82)	1/28(3)	28/29(97)	6/6(100)	4/4(100)	8/8(100)	15/15(100)	7/7(100)	2/2(100)
**PD‐L1 (Hscore)**										
0	79/109(72)	1/12(8)	20/26(77)	24/27(89)	6/7(86)	3/4(75)	3/7(43)	13/16(81)	7/8(87)	2/2(100)
1–100	21/109(19)	5/12(42)	6/26(23)	2/27(7)	–	1/4(25)	4/7(57)	2/16(12)	1/8(12)	–
101–200	4/109(4)	2/12(16)	–	–	1/7(14)	–	–	1/16(6)	–	–
201–300	4/109(4)	4/12(33)	–	–	–	–	–	–	–	–
**PD1 (Hscore)**										
0	94/108(87)	11/11(100)	25/27(92)	25/25(100)	7/7(100)	3/4(75)	8/8(100)	8/16(50)	6/8(75)	1/2(50)
1–100	14/108(13)	–	2/27(7)	–	–	1/4(25)	–	8/16(50)	2/8(25)	1/2(50)
101–200	–	–	–	–	–	–	–	–	–	–
201–300	–	–	–	–	–	–	–	–	–	–
**CD30**										
Negative	85/110(77)	5/12(42)	21/26(81)	19/26(73)	7/7(100)	4/4(100)	8/10(80)	13/16(81)	7/7(100)	1/2(50)
Positive	25/110(23)	7/12(58)	5/26(19)	7/26(27)	0/7(0)	0/4(0)	2/10(20)	3/16(19)	0/7(0)	1/2(50)
**Genes**										
*MYD88*	52/113(45)	1/12(8)	13/28(46)	19/26(73)	4/7(57)	3/4(75)	2/10(20)	8/16(50)	1/8(12)	0/2(0)
*CD79B*	45/113(40)	1/12(8)	14/28(50)	15/26(58)	4/7(57)	2/4(50)	1/10(10)	6/16(37)	1/8(12)	1/2(50)
*CARD11*	20/111(18)	2/12(16)	7/28(25)	7/24(29)	0/7(0)	0/4(0)	3/10(30)	1/16(6)	0/8(0)	0/2(0)
*BTK*	4/111(4)	0/12(0)	1/28(3)	0/24(0)	0/7(0)	1/4(25)	1/10(10)	1/16(6)	0/8(0)	0/2(0)

Abbreviations: CNS, central nervous system; ENT, ear nose and throat; IPI, International prognostic index; PD, primary diagnosis.; PMBL, Primary mediastinal B‐cell lymphoma.

### 3.2 Immunohistochemistry (IHC)

**FIGURE 1:**
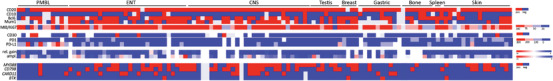
Immunohistochemical, FISH, and sequencing data across different extranodal localization. Heatmap indicating the IHC data for CD20, CD10, Bcl6, Mum1, CD30, PD1, PD‐L1, and FISH data for relative gain and amplification of the PDL1/2 locus and the sequencing data for *MYD88*, *CD79B*, *CARD11*, and *BTK*

Subtyping of the lymphomas using CD20, CD10, Bcl2, and MUM1 revealed a total of 27/99 (27%) germinal center B‐cell (GCB) and 73/99 (74%) non‐GCB lymphomas according to the Hans classifier. Non‐GCB subtype was predominant across all sites, with the lowest proportion of this subtype found in bone (28%). Expression of PD‐L1, as measured by the H‐score, varied depending on the location of extranodal DLBCL and showed statistically significant higher expression levels in PMBL (*p* = 0.0001) compared with all other sites. H‐scores ranged from 1 to 300 (Figure 1, Table 1). Besides PMBL, with a PD‐L1 expression of 11/12 (92%), relatively high proportions of PD‐L1‐expressing extranodal DLBCL were found in the gastric region, ENT, and skin (57%, 23%, and 18%, respectively). PD1 expression was seen in DLBCL localized in skin (50%), breast (25%), bone (25%), and ENT (7%). CD30 expression was seen in PMBL (58%), DLBCL in the CNS (27%), skin (19%), and ENT (19%). The results in PMBL were similar to those of PD‐L1, showing a significantly higher expression of CD30 compared with the other sites (*p* = 0.0052).

### 3.3 Epstein Barr Virus (EBV)

Only three of 109 (2%) EBV‐positive cases were found: two PMBL and one PE‐DLBCL of the CNS.

### 3.4 Fluorescence in situ hybridization (FISH)

The highest percentage of 9p24.1 alterations was found in relation to the relative gain of the *PDL1/2* locus in 57% (59/99) of cases. The percentage of altered tumor cells in relation to the *PDL1/2* locus ranged from 29% (2/7) in gastric DLBCL to 61% (14/23) in CNS lymphoma and 75% (3/4) in bone extranodal DLBCL. Amplification with more than 15% of cells was found in only two of 99 (2%), comprising one PMBL and one extranodal DLBCL of the ENT. The second most common aberration was polysomy in 22 of 99 (22%) of cases; percentages varied from 33% (1/3) in DLBCL of breast up to 43% (3/7) in gastric lymphoma. We also found a relative loss of the *PDL1/2* region to be most prevalent in extranodal DLBCL of the stomach (2/7; 29%) and testis (2/7; 29%), followed in decreasing order by ENT (5/25; 20%), skin (3/16; 19%), CNS (2/23; 9%), and finally PMBL (1/12; 8%). No aberrations with respect to the *PDL1/2* locus could be found in two of the tested cases (2/99; 2%). Both cases were extranodal ENT lymphomas (Table 2).

**TABLE 2:** Results of fluorescence in situ hybridization of the *PDL1/2* locus

Alteration	Total *N* = 116	PMBL *N* = 12	ENT *N* = 28	CNS *N* = 29	Testis *N* = 7	Breast *N* = 4	Stomach *N* = 10	Skin *N* = 16	Bone *N* = 8	Spleen *N* = 2
**Polysomy**	22/99 (22)	4/12 (33)	4/25 (16)	5/23 (22)	1/7 (14)	1/3 3(3)	3/7 (43)	3/16 (19)	1/4 (25)	–
**Relative gain**	59/99 (57)	6/12 (50)	15/25 (60)	14/23 (61)	4/7 (57)	2/3 (67)	2/7 (29)	10/16 (63)	3/4 (75)	2/2 (100)
**Amplification**	2/99 (2)	1/12 (8)	1/25 (4)	–	–	–	–	–	–	–
**Relative loss**	15/99 (15)	1/12 (8)	5/25 (20)	2/23 (9)	2/7 (29)	–	2/7 (29)	3/16 (19)	–	–
**No aberration**	2/99 (2)	–	–	2/23 (9)	–	–	–	–	–	–

Abbreviations: CNS, central nervous system; ENT, ear, nose, and throat; PMBL, Primary mediastinal B‐cell lymphoma.

Importantly, no correlation was seen between PD‐L1 expression and *PD‐L1/2* gains/amplifications (Figure S1).

### 3.5 Mutational status

Molecular analysis of *MYD88*, *CARD11*, *CD79B*, and *BTK* was performed in 113 patients (97%). *MYD88* mutations were identified in 51 (45%) cases, of which 44 harbored the hotspot L256P mutation, and the sole mutation detected in 16/113 (14%). Apart from this hotspot mutation, five further mutations in *MYD88* (S251N, R239Q, D148H, P258L, and S219C) were detected. However, the impact of these mutations is unknown due to their low prevalence. A high incidence of *MYD88* L256P mutations was detected in DLBCL in the breast (3/4; 75%), CNS (16/26; 62%), testis (4/7; 57%), ENT (12/28; 43%), and skin (7/16; 44%). Forty‐five (40%) lymphomas had a *CD79B* mutation, including three cases with the Y196 hotspot mutation, and 26 lymphomas had a Y197 mutation. The other *CD79B* mutations detected were localized at S72P, G190D, M64L, E93K, E198G, L200P, L200Q, M164I, E192Q, M14I, and D182E. *CD79B* was frequently mutated in DLBCLs in the testis (57%), CNS (58%), breast (50%), ENT (50%), spleen (50%), and skin (37%). Thirty‐four of 113 (30%) lymphomas harbored both *MYD88* and *CD79B* mutations and were found in the breast (2/4; 50%), CNS (13/26; 50%), ENT (10/28; 36%), skin (5/16; 31%), testis (2/7; 28%), stomach (1/10; 10%), and PMBL (1/12; 8%). Disregarding cases with *CD79B* mutations, 17/113 (15%) showed a *MYD88* mutation alone. This was true for 6/26 (23%) of CNS lymphomas, 2/7 (28%) located in the testis, 1/4 (25%) in the breast, 3/16 (19%) in the skin, 1/8 (12%) in the bone, 1/10 (10%) the stomach, and 3/28 (11%) in the ENT. For *CD79B*, irrespective of *MYD88* mutational status, mutations were found in 4/28 (14%) of ENT lymphomas, 2/26 (8%) of CNS lymphomas, 2/7 (28%) of lymphoma of the testis, 1/8 (12%) of bone lymphomas, 1/2 (50%) of lymphomas located in the spleen, and 1/16 (6%) of skin lymphomas. *CARD11* mutations were detected in 20 lymphomas. *CARD11* mutations occurred frequently in gastric lymphoma (3/10; 30%) but also in ENT (7/28; 25%), CNS (7/24; 29%), and skin (1/16; 6%) lymphomas. *BTK* mutations were uncommon and detected in only four lymphomas: breast, skin, ENT, and stomach (Figure 1, Table 1, for allele frequencies, specific mutations, and tumor cell see Tables S2 and S3).

Older patients (age >60 years) had a higher proportion of *MYD88* mutations (*p* = 0.0036), and either a mutated *MYD88* or *CD79B* (*p* = 0.0034). There was no correlation between age and *CD79B* mutation or age and both *MYD88* and *CD79B* mutations.

### 3.6 Immunohistochemistry of mutated cases

As mentioned above, a total of 51/113 (45%) of the cases harbored a mutation of the *MYD88* gene (including 5 “other than L265P mutations”). PD‐L1 expression with an H‐score >1 was found in 8/51 (16%) of the mutated cases harboring a *MYD88* mutation and co‐expressing PD‐L1, with only two cases in each of the CNS, ENT, and skin, one case of PMBL, and one gastric lymphoma. On a genomic level, 14 of 45 (31%) showed a relative gain of the *PDL1/2* locus, whereas two of 45 (4%) were amplified.

Furthermore, *MYD88* and/or *CD79B* mutations are often associated with copy gains on 9p24.1 but not with expression of PD‐L1 detected by immunohistochemistry (Figures S4–S6).

### 3.7 Survival analysis

There was a significant difference in the overall survival (OS) (*p* = 0.004) and progression free survival (PFS) (*p* = 0.01) in patients with International Prognostic Index (IPI) status <1 in comparison with IPI status >1 (Figure 2A). Patients over the age of 60 had a significantly lower OS (*p* = 0.01) and PFS (*p* = 0.05) (Figure 2B) in comparison with younger patients. Higher ECOG (performance status of the eastern cooperative oncology group) values had a significantly poor OS (*p* = 0.000007) and PFS (*p* = 0.00002) when compared with lower ECOG values (Figure 2C). The differences in PFS and OS between GCB and non‐GCB lymphomas were not significant (Figure 2D). A significant difference in PFS (*p* = 0.006) and OS (*p* = 0.010) was detected between DLBCLs according to the site (Figure 2E).

FIGURE 2Survival analysis. Kaplan–Meier plot indicating differences in OS and PFS based on the International Prognostic Index (IPI) status (A), age (B), ECOG (C), GCB versus non‐GCB (D), localization (E), and mutational status of *MYD88* (F), *CD79B* (G), and *BTK(H)*

**Figure:**
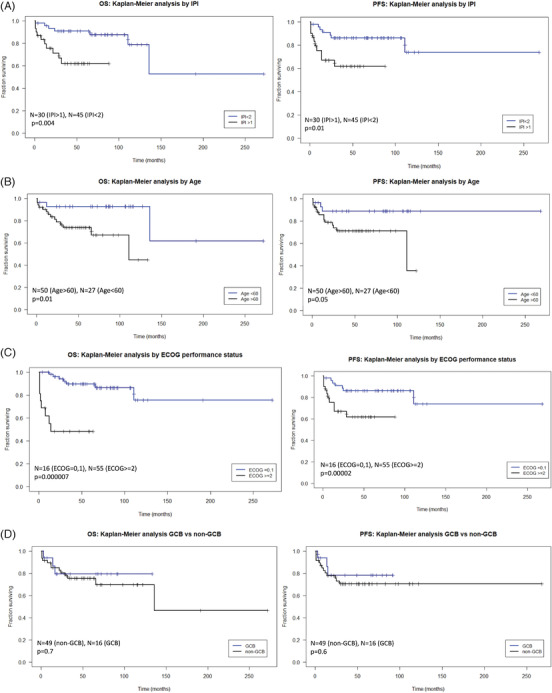

**Figure:**
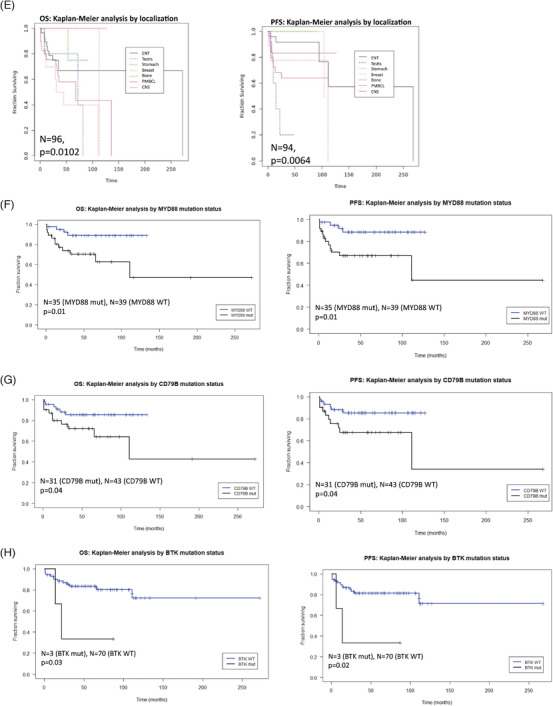

There was a significantly lower OS (*p* = 0.01) and PFS (*p* = 0.01) in patients with a *MYD88* mutation than in patients with *MYD88* wild type (WT) (Figure 2F). Similarly, significantly reduced OS (*p* = 0.04) and PFS (*p* = 0.04) were observed for the *CD79B* mutated lymphomas in comparison with patients with *CD79B* WT (Figure 2G). The prognostic impact of *MYD88* and *CD79B* mutations vanished if CNS lymphomas were excluded (*p* = 0.2 and 0.6, respectively; Figure S2). Patients with mutated *BTK* had a significantly lower OS (*p* = 0.03) and PFS (*p* = 0.02) than patients with *BTK* WT; however, the small number of only four mutated cases has to be taken into account (Figure 2H).

For immunohistochemistry, we analyzed OS for extranodal DLBCL, excluding PMBL, and found no statistically significant difference between PD‐L1 positive and negative cases (*p* = 0.7). The same was true for CD30 when excluding PMBL (*p* = 0.6) (Figure S3).

## 4 DISCUSSION

We described the prevalence and prognostic impact of targetable alterations in PE‐DLBCLs. As a main finding, targetable alterations are more common than expected in DLBCLs not specifically selected for a PE origin. In this single‐center analysis, we collected extranodal biopsy samples from homogeneously treated patients with DLBCLs of limited disease.

As expected, non‐GCB cases were more frequent in all subentities, with the exception of DLBCL manifesting only in bone lesions. These patients were younger than the rest of our cohort (six of eight patients ≤65 years of age). This exceptional position of bone lymphoma has been described previously [20, 21]. Targetable alterations are scarce in bone lymphoma; however, in contrast to previous publications [22], we identified one case with a *MYD88* and one case with a *CD79B* mutation.

PD‐L1 expression is a hallmark in Hodgkin lymphoma, but also in PMBL [23, 24] and in some cases of CNS and testicular lymphoma [12], and might be predictive for the checkpointinhibitor (CPI) response. Regarding PD‐L1 expression in PMBL, we found expression in 96% of our cases, which is in line with published data [25, 26]. On the contrary, PD‐L1 expression is infrequent (9%) in nonselected DLBCL patients [27]. We identified a higher rate of PD‐L1 expression in gastric, ENT, and skin lymphomas (57%, 23%, and 18%, respectively). The same is true for copy gains in these entities compared with the prevalence in nonselected DLBCLs (16%) [27]. We did not see any influence of PD‐L1 expression on the PFS or OS in our cohort. However, there are reports stating that tumor cells expressing PD‐L1 may represent a biomarker for poor prognosis [28, 29]. ENT lymphomas express PD‐L1 more frequently than previously realized and thus could be treatable with CPI. PD‐L1 expression and 9p24.1 gains were significantly lower in the CNS lymphomas of our cohort than in the published data [30]. The number of gains or amplification on 9p21.1 does not correlate with expression of PD‐L1. This finding has been confirmed by other studies [31]. Since there are ongoing studies analyzing expression of PD‐L1 in *MYD88* mutated cases to predict outcome [32], we correlated those parameters and found a PD‐L1 expression in 16% of the *MYD88* mutated cases, with distribution throughout the entire cohort, but with no significant differences in OS (*p* = 0.5) or PFS (*p* = 0.5). CD30 expression was seen in PMBL, lymphomas of the spleen, CNS, skin, and ENT (58%, 50%, 27%, 19%, and 19%, respectively). These lymphomas could possibly be targeted by brentuximab vedotin therapy, which consists of a CD30‐directed antibody. Targeting CD30 is shown to be a feasible regimen in CD30‐expressing B‐cell lymphomas, including PMBL [15]. There are also conflicting reports on the prognostic value of CD30 in DLBCL [8]. However, in our analysis, we did not observe any influence of CD30 expression on survival. This has also been reported by Salas et al. [33] In the literature, PMBL is mostly described as negative for EBV [34]. In our cohort, we detected 18% PMBLs with concomitant EBV positivity. In the literature, an EBV infection in classical Hodgkin lymphoma represents an alternative mechanism of PD‐L1 induction [35]. Two of the PMBLs with EBV infection also showed PD‐L1 positivity, but only one of them had a relative gain on a genomic level as detected by FISH.

*MYD88* and *CD79B* mutations have a higher incidence in certain extranodal lymphomas (CNS, skin, and breast) [3]. A high incidence of *MYD88* and *CD79B* mutations was also detected in the ENT lymphomas in our cohort. This is not in line with previous findings, since Ollila et al. reported a lack of *MYD88* in craniofacial DLBCL [3]. *CARD11* mutations occur frequently in gastric lymphomas (30%) and ENT lymphomas (25%) and might explain BTKi resistance [36]. The *MYD88* L265P mutation and the *CD79B* Y196 mutation in DLBCL are significantly associated with the non‐GCB subtype. These mutations play a key role in B‐cell receptor (BCR) activation, providing a survival advantage [37, 38]. Other studies have likewise reported that *MYD88* L265P occurs at a significantly higher frequency in the non‐GCB subtype [39, 40, 41]. Furthermore, *MYD88* L265P frequently co‐occurs in DLBCL harboring a *CD79B* mutation, indicating synergistic effects on BCR signaling [42].

A significant difference can be seen in the PFS and OS correlation with respect to localization of the DLBCL, and there was a significant difference in OS and PFS regarding the IPI status, which is in line with previous reports [43]. Regarding PFS and OS related to the lymphoma subtype or cell of origin, determined with the Hans classifier, we could not detect any significant differences. *MYD88, CD79B*, and/or *BTK* mutated lymphomas had a lower OS and PFS, which can mostly be attributed to the group of primary CNS lymphomas. However, there is a tendency to a lower OS in patients with *MYD88* mutations than in patients with lymphomas from other sites, which is in line with the poor outcome of patients from “cluster 5” or MCD type. Vermaat et al. recently published similar findings in relation to *MYD88* and *CD79B* mutations in a large cohort of DLBCL including 108 patients with extranodal DLBCL [44]. Concerning the mutational status, they report only 14.8% *MYD88* mutated cases. In our cohort, however, *MYD88* mutations occurred in 45% of all cases irrespective of *CD79B* mutations, while 15% showed an *MYD88* mutation alone. The same was true for *CD79B*, as they found only 9.3% of the cases had mutated, but we detected this mutation in about 40% of our cases irrespective of the *MYD88* mutational status, and 9% with this mutation alone. Thirty percent of the lymphomas in our cohort harbored both *MYD88* and *CD79B* mutations. We therefore observed a higher incidence of those mutations with respect to PE‐DLBCL than found in the published data. Furthermore, the extranodal DLBCLs harboring both the *MYD88* and *CD79B* mutation could possibly be targeted by ibrutinib along with R‐CHOP [10]. In addition to the therapies mentioned, it is also worth mentioning that polatuzumab, an antibody against CD79b, can be used in DLBCL [45]. Pfeifer et al. could show that *CD79B* mutations do not appear to affect the efficacy of polatuzumab in vitro. However, it is not yet clear what impact a mutation in *CD79B* has in vivo [46].

In summary, our data underline the clinical and biological similarity of lymphomas of PE origin. We identified a high number of alterations, which might be predictive for modern targeted treatment strategies. These data emphasize that refractory or relapsed PE‐DLBCLs should be evaluated for these aberrations and discussed in molecular tumor boards. Since many phase III “all comers” trials of novel drugs within the last decade have failed, a focus on PE‐DLBCL might help to establish new treatment strategies in patients with unmet medical needs.

## CONFLICT OF INTEREST

All authors declare that they have no conflict of interest.

## AUTHOR CONTRIBUTIONS

Stephanie E. Weissinger and Andreas Viardot designed the study. Stephanie E. Weissinger, Rucha Dugge, Miriam Disch, and Malena Zahn performed experiments. Rucha Dugge and Johannes Bloehdorn performed calculations. Stephanie E. Weissinger and Peter Möller analyzed data. Andreas Viardot, Rucha Dugge, Thomas F. Barth, and Stephanie E. Weissinger wrote the manuscript. Andreas Viardot and Peter Möller supervised the findings.

## ETHICS STATEMENT

The authors declare that the study was approved by the local ethics committee (application number: 381/17) and was conducted in accordance with the Declaration of Helsinki.

## Supporting information

Supporting Information

Supporting Figure

Supporting Table

Supporting Table

Supporting Information

## Data Availability

Raw data were generated at the Institute of Pathology, Ulm, Germany and are available from the corresponding author PM on reasonable request.
