# Supplementary material for: Targetable alterations in primary extranodal diffuse large B‐cell lymphoma
Source: EJHaem. 2022 May 23;3(3):688–97. doi: 10.1002/jha2.428 (PMC9421950; doi:10.1002/jha2.428)
Supplement: Supplementary file 1 — Supporting Information [file JHA2-3-688-s002.docx]

**Supplemental information methodology**

*Tissue specimens and immunohistochemical analysis*

FFPE tissue specimens were obtained during routine diagnostic procedures at the Ulm University Hospital, Ulm, Germany. All biopsies were representative of the tumor core. Immunohistochemical staining (IHC) was performed on a Dako Omnis Autostainer (Dako, Glostrup, Denmark) using the manufacturer’s optimized protocol for each antibody (Supplementary Table 1). All antibodies were then visualized with Dako REAL^TM^ Chromogen Red; hematoxylin (Dako, Glostrup, Denmark) was used for counterstaining.

For analysis, whole slides were considered independent of tissue size. A cut-off of >30% was chosen for CD20, CD10, Mum1, and Bcl6 positivity, concordant to the Hans classifier, which was also used for determining the germinal center B-cell (GCB) or non-GCB subtype(1). A modified H-score was used to evaluate PD1 and PD-L1(2). CD30 positivity was defined as >1% of tumor cells expressing CD30(3).

*CISH*

For EBV analysis, the ZytoFast^R^ EBV-CISH Kit (Zytovision, Germany) was used.

*Fluorescence in situ hybridization (FISH)*

For FISH genetic analysis, 5 µm thick FFPE tissue sections were used for hybridization. We assessed gene copy number variations of the PDL1/2 gene on chromosome 9p24.1 (SPEC CD274/CEN9 dual color probe, PDCD1LG2, Zytovision, Zytomed Systems, Berlin, Germany). The probe was assessed in regions with at least 50 nuclei. Evaluation was performed in accordance with previous publications(4). Green signal (g): *PDL1/2* locus, red signal (r): *CEN9*; 2g2r was defined as disomy, >2g2r (1:1) as polysomy, >1g1r but <3g1r as relative copy gain, ≥3g1r as amplification, >1r1g but <3r1g as relative copy loss, and ≥3r:1g as loss. The cases were screened on single cell level for aberrations, dividing the single cells into ‘normal’ (disomy), those with relative gain or amplification, relative loss, loss, and polysomy. Results are depicted in altered nuclei per screened cells in percent (%). In each case, the percentage and magnitude of 9p24.1 amplification, copy gain, polysomy, and normal copy numbers (disomy) was assessed and each case was classified by the highest observed level of 9p24.1 aberration.

*Mutational analysis (NGS lymphoma panel)*

FFPE tissue DNA was used for amplicon sequencing. DNA was isolated using the Qiamp DNA Mini Kit (Qiagen, Hilden, Germany). Up to 20 ng DNA was used for each library. The library was prepared using the lymphoma panel (Qiagen, Hilden, Germany), which allows the sequencing of *MYD88, CD79B, CARD11,* and *BTK* genes. Prepared libraries were sequenced by targeted next-generation sequencing on a MiSeq platform (Illumina, San Diego, CA, USA) with paired-end 150-base pair reads (approximately 5000x coverage). Fastq files were uploaded to Qiagen CLC Biomedical Workbench V5.2 (Hilden, Germany). Further samples were analyzed using ready-to-use workflow ‘Identify and Add Variants’ and reads were mapped against Human Genome Build 19 (hg19) as the reference. In addition, the Integrative Genomics Viewer (IGV) was used to visualize variants.

*Generating heatmaps and Kaplan-Meier curves*

Heatmaps were generated using the python programming language(5) (version 3.8.8), extended by matplotlib (version 3.3.4), numpy (version 1.19.5), and pandas (version 1.2.2) packages. Kaplan-Meier curves were generated using RStudio (version 4.1.1)(6) with survival package (version 3.2-13)(7).

*Statistical analysis*

Progression-free survival (PFS) and overall survival (OS) were defined as the time from start of treatment to disease progression or death and death from any cause, respectively. PFS and OS were determined and survival analysis performed with Kaplan-Meier curves. Differences between groups were assessed using two-sided non-stratified log-rank tests. Statistical software: Statistical analysis was performed with MATLAB (MathWorks, Natick, MA), R version 3.4.1, SPSS version 21 (IBM, NYC, NY) and Prism software version 6.0 (GraphPad).

**References**

1. Hans CP, Weisenburger DD, Greiner TC, Gascoyne RD, Delabie J, Ott G, et al. Confirmation of the molecular classification of diffuse large B-cell lymphoma by immunohistochemistry using a tissue microarray. Blood. 2004;103(1):275–82.

2. Stenger M. Calculating H-Score. The ASCO Post. 2015.

3. Jacobsen ED, Sharman JP, Oki Y, Advani RH, Winter JN, Bello CM, et al. Brentuximab vedotin demonstrates objective responses in a phase 2 study of relapsed/refractory DLBCL with variable CD30 expression. Blood. 2015 Feb;125(9):1394–402.

4. Roemer MGM, Advani RH, Ligon AH, Natkunam Y, Redd RA, Homer H, et al. PD-L1 and PD-L2 Genetic Alterations Define Classical Hodgkin Lymphoma and Predict Outcome. J Clin Oncol Off J Am Soc Clin Oncol. 2016 Aug;34(23):2690–7.

5. van Rossum G. Python tutorial, May 1995. CWI Rep CS-R9526. 1995;(CS-R9526):1–65.

6. R: The R Project for Statistical Computing [Internet]. [cited 2021 Dec 15]. Available from: https://www.r-project.org/

7. Therneau TM, until 2009) TL (original S->R port and R maintainer, Elizabeth A, Cynthia C. survival: Survival Analysis [Internet]. 2021 [cited 2021 Dec 15]. Available from: https://CRAN.R-project.org/package=survival
