# Supplementary material for: Targetable alterations in primary extranodal diffuse large B‐cell lymphoma
Source: EJHaem. 2022 May 23;3(3):688–97. doi: 10.1002/jha2.428 (PMC9421950; doi:10.1002/jha2.428)
Supplement: Supplementary file 2 — Supporting Figure [file JHA2-3-688-s004.pptx]

## Slide 1
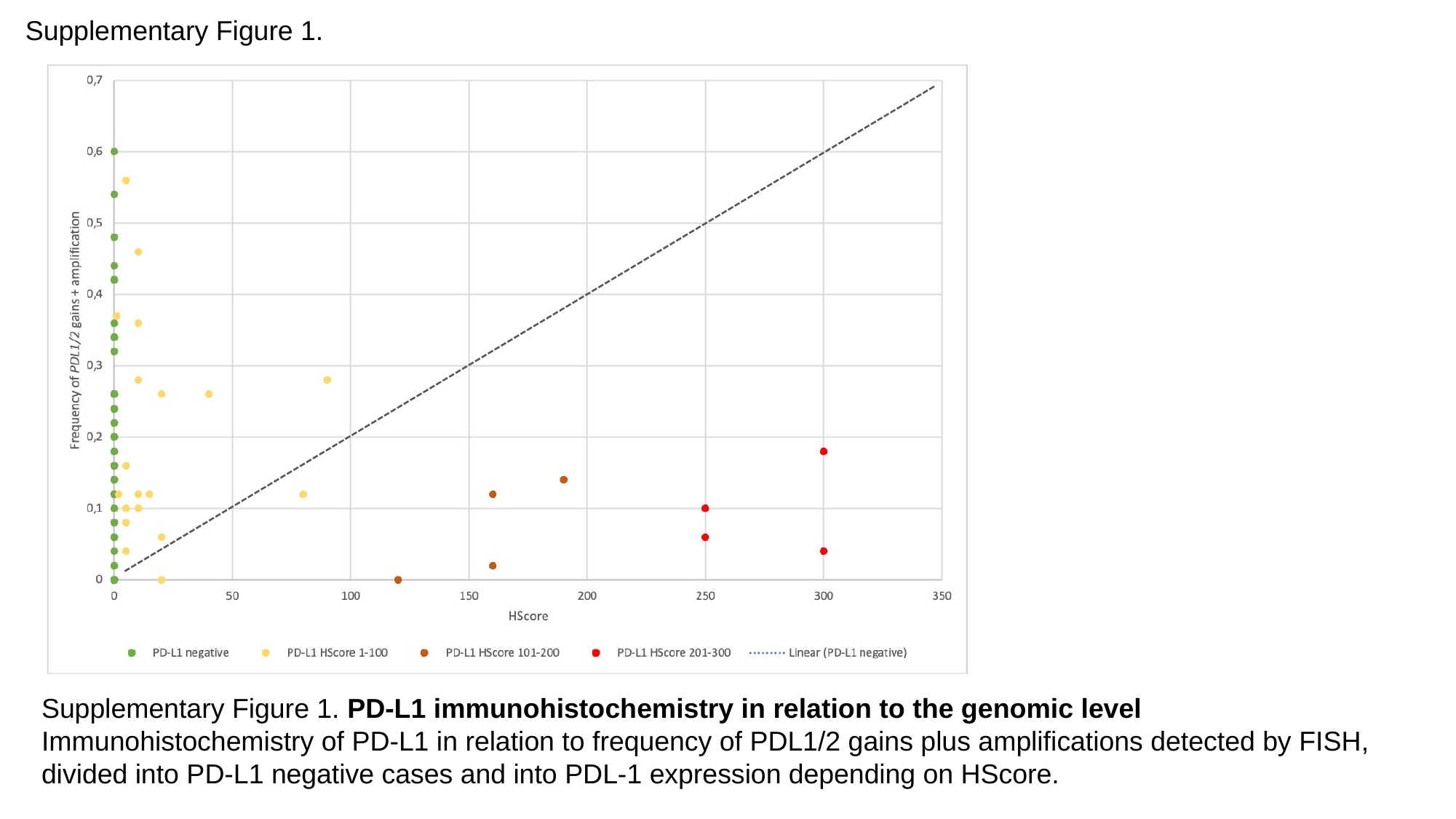

Supplementary Figure 1.
Supplementary Figure 1. PD-L1 immunohistochemistry in relation to the genomic level
Immunohistochemistry of PD-L1 in relation to frequency of PDL1/2 gains plus amplifications detected by FISH, divided into PD-L1 negative cases and into PDL-1 expression depending on HScore.

## Slide 2
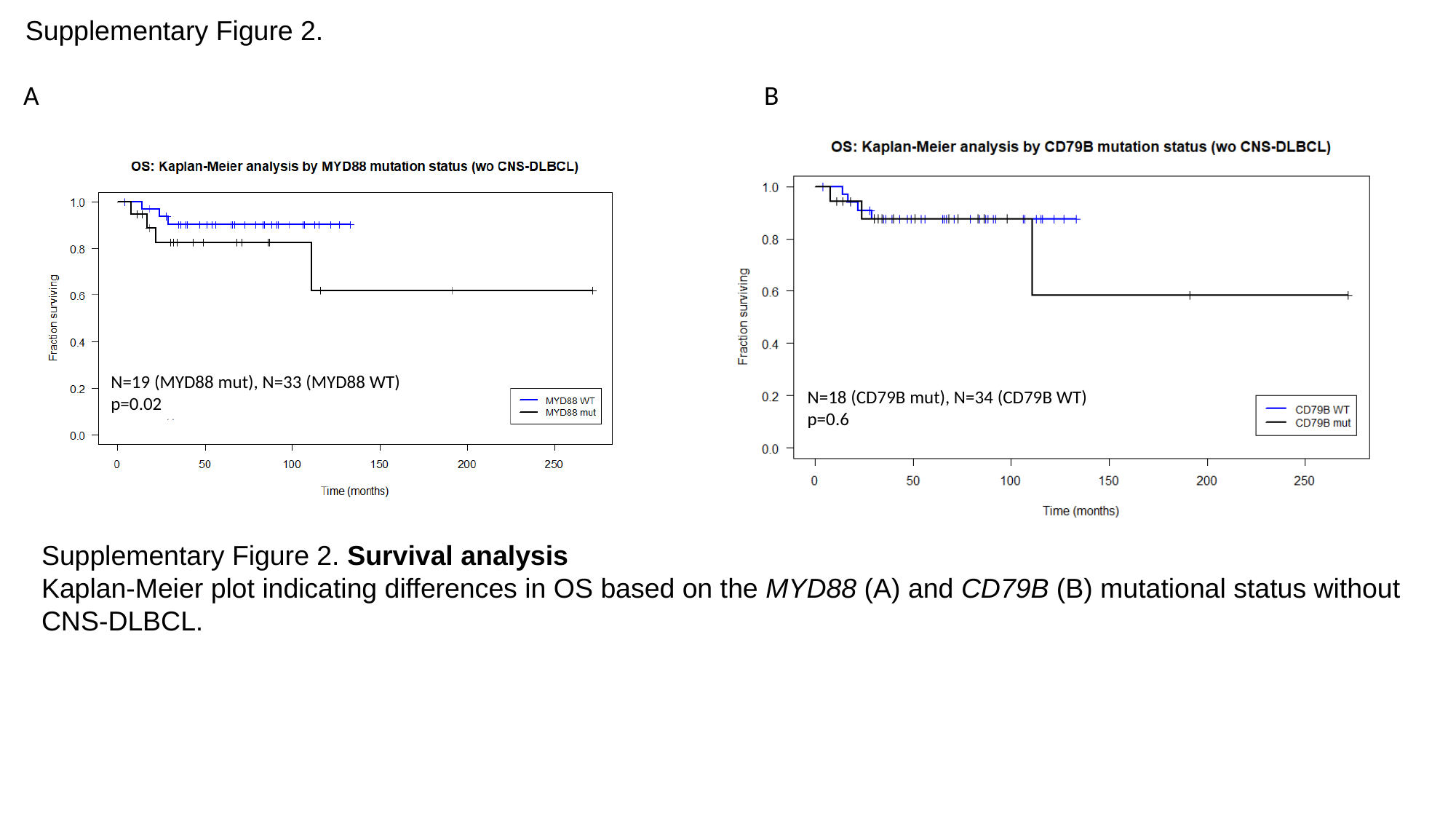

Supplementary Figure 2.
A
B
N=19 (MYD88 mut), N=33 (MYD88 WT)
p=0.02
N=18 (CD79B mut), N=34 (CD79B WT)
p=0.6
Supplementary Figure 2. Survival analysis
Kaplan-Meier plot indicating differences in OS based on the MYD88 (A) and CD79B (B) mutational status without CNS-DLBCL.

## Slide 3
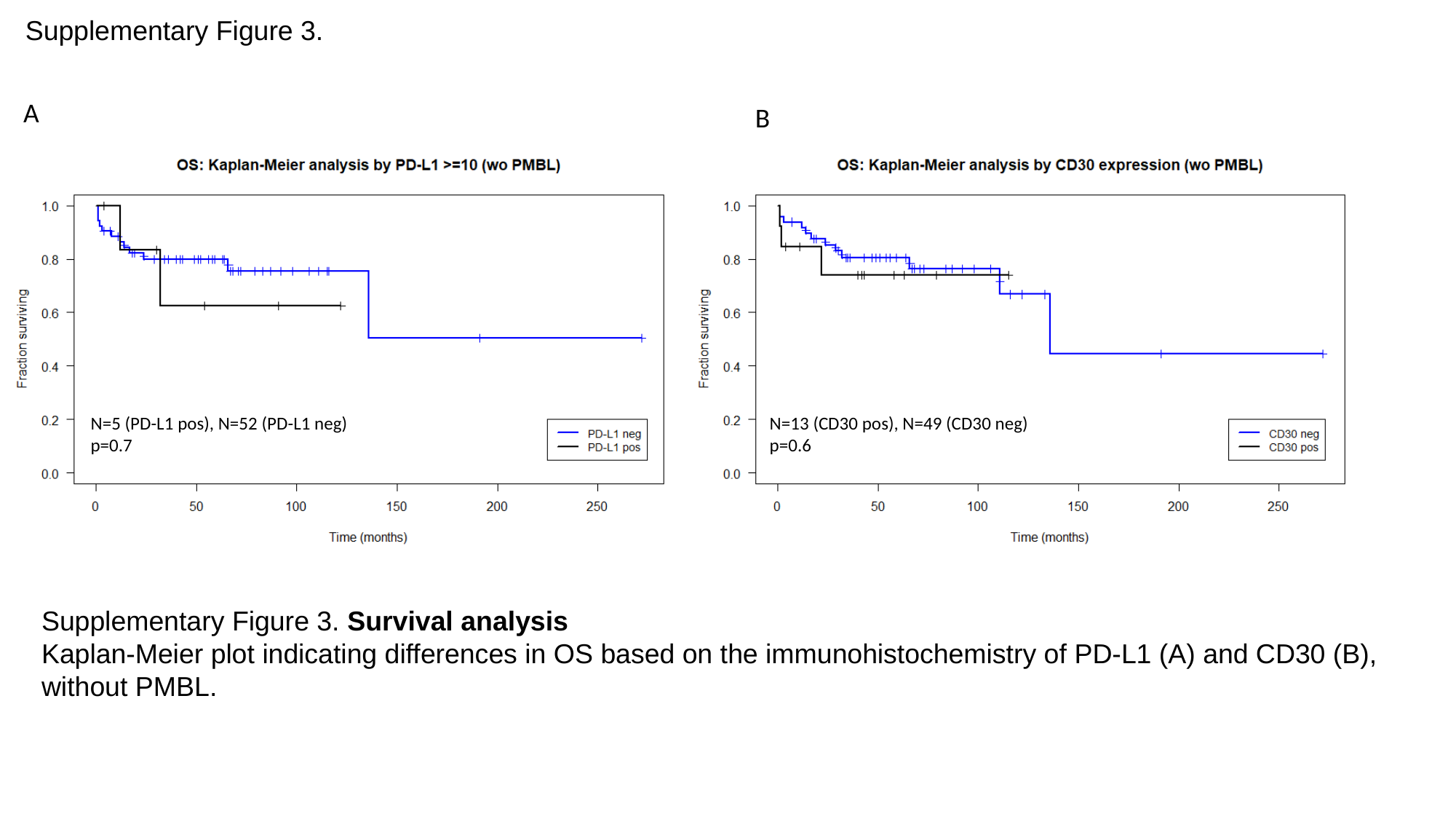

Supplementary Figure 3.
A
B
N=5 (PD-L1 pos), N=52 (PD-L1 neg)
p=0.7
N=13 (CD30 pos), N=49 (CD30 neg)
p=0.6
Supplementary Figure 3. Survival analysis
Kaplan-Meier plot indicating differences in OS based on the immunohistochemistry of PD-L1 (A) and CD30 (B), without PMBL.

## Slide 4
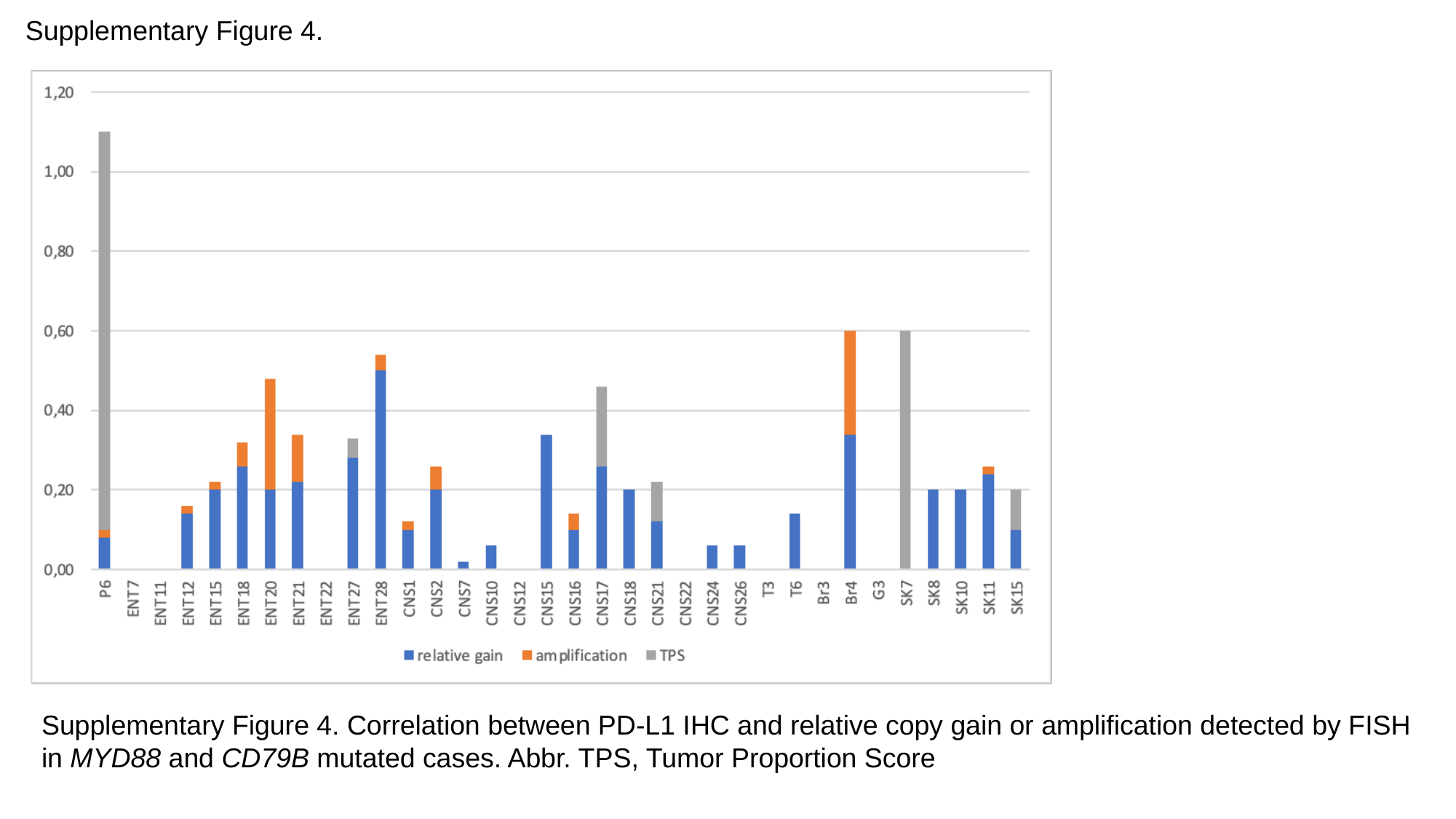

Supplementary Figure 4.
Supplementary Figure 4. Correlation between PD-L1 IHC and relative copy gain or amplification detected by FISH in MYD88 and CD79B mutated cases. Abbr. TPS, Tumor Proportion Score

## Slide 5
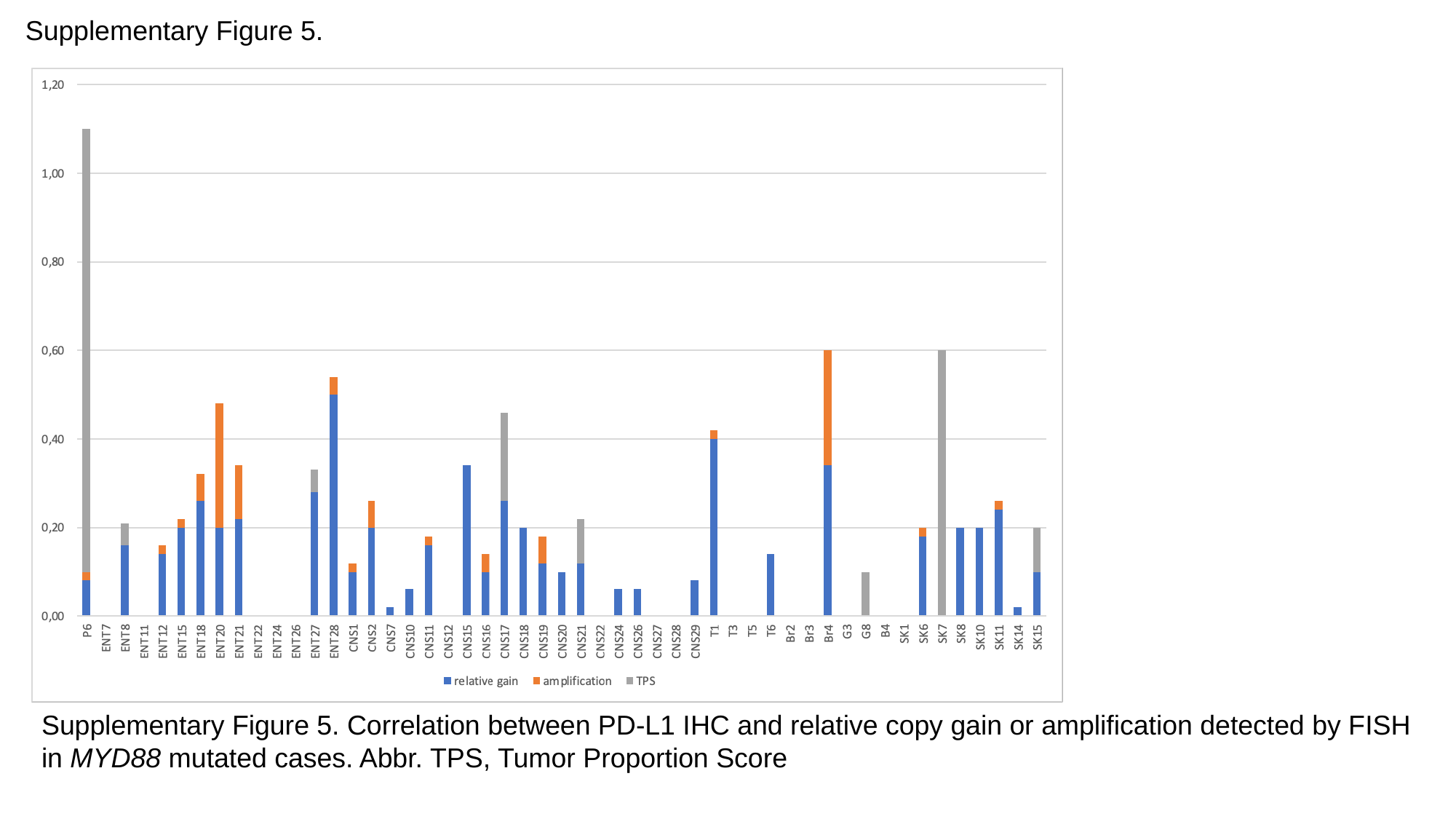

Supplementary Figure 5.
Supplementary Figure 5. Correlation between PD-L1 IHC and relative copy gain or amplification detected by FISH in MYD88 mutated cases. Abbr. TPS, Tumor Proportion Score

## Slide 6
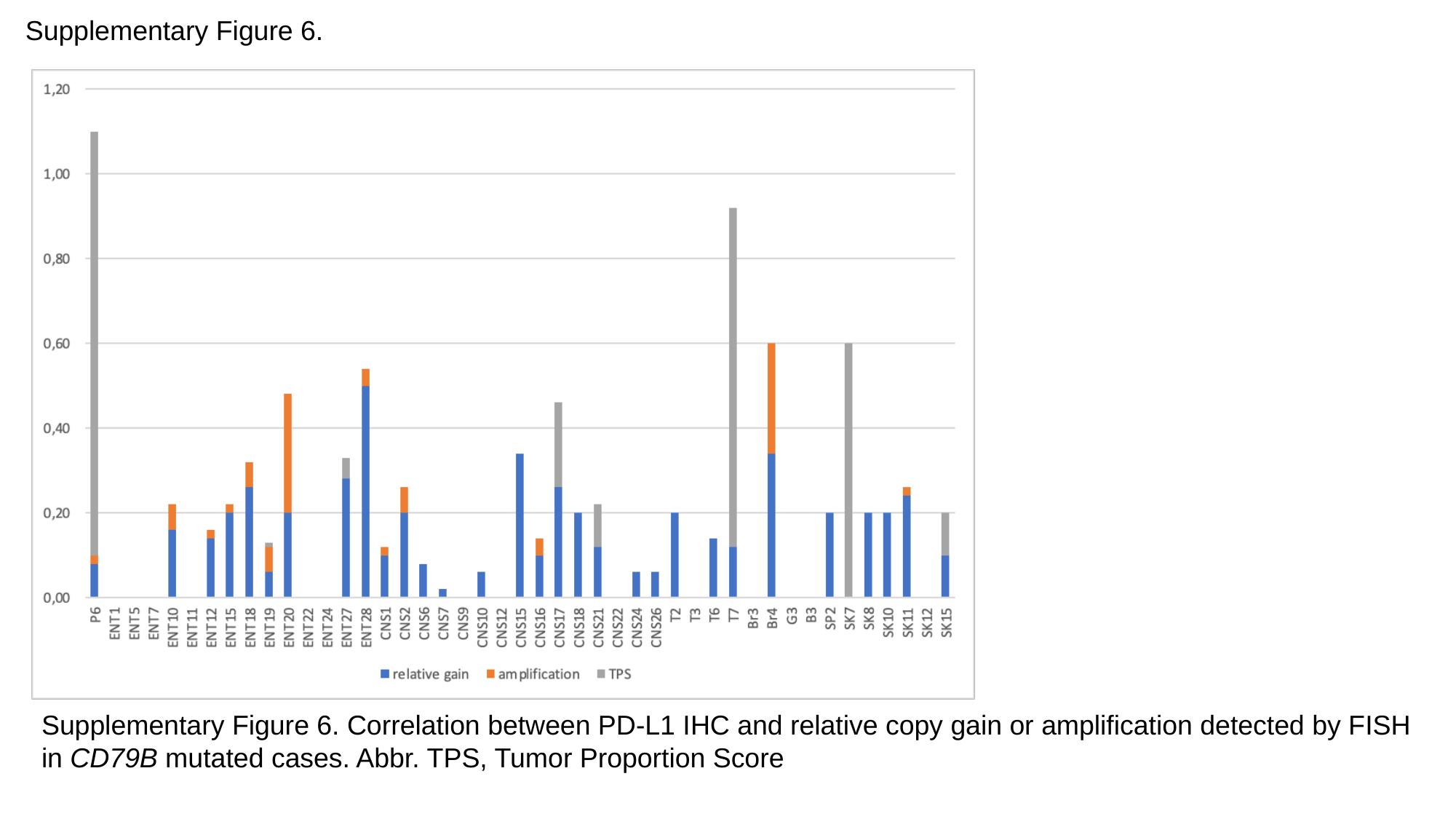

Supplementary Figure 6.
Supplementary Figure 6. Correlation between PD-L1 IHC and relative copy gain or amplification detected by FISH in CD79B mutated cases. Abbr. TPS, Tumor Proportion Score
