# Supplementary material for: Targetable alterations in primary extranodal diffuse large B‐cell lymphoma
Source: EJHaem. 2022 May 23;3(3):688–97. doi: 10.1002/jha2.428 (PMC9421950; doi:10.1002/jha2.428)
Supplement: Supplementary file 3 — Supporting Table [file JHA2-3-688-s005.docx]

**Supplementary Table 1**. Antibody details used for immunohistochemical staining

| *antibody* | *company* | *clone/cat.no* | *dilution* | *pretreatment* | *staining pattern* |
| --- | --- | --- | --- | --- | --- |
|  |  |  |  |  |  |
|  |  |  |  |  |  |
| CD20 | Agilent/Dako, USA | L26 | rtu | high | mem |
| CD10 | Agilent/Dako, USA | 56C6 | rtu | high | mem |
| Bcl6 | Agilent/Dako, USA | PG-B6p | rtu | high | nuc |
| MUM1 | Agilent/Dako, USA | MUM1p | rtu | low | nuc |
| CD30 | Agilent/Dako, USA | Ber-H2 | rtu | low | mem |
| MIB1/Ki67 | Agilent/Dako, USA | MIB | rtu | high | nuc |
| PD1 | Dianova, Germany | JAD1 | 1:200 | high | mem |
| PD-L1 | Quartett, Germany | QR1 | 1:50 | Tris/EDTA (pH9) | mem |
| LMP | Agilent/Dako, USA | Cs1-4 | rtu | high | nuc |
|  |  |  |  |  |  |
|  |  |  |  |  |  |

**Abbreviations**: mem, membranous; nuc, nuclear; rtu, ready-to-use
