# Supplementary material for: Targetable alterations in primary extranodal diffuse large B‐cell lymphoma
Source: EJHaem. 2022 May 23;3(3):688–97. doi: 10.1002/jha2.428 (PMC9421950; doi:10.1002/jha2.428)
Supplement: Supplementary file 4 — Supporting Table [file JHA2-3-688-s003.docx]

**Supplementary Table 3**. Results of mutational profiling.

|  |  |  | |  | |  |  |  |  |  |
| --- | --- | --- | --- | --- | --- | --- | --- | --- | --- | --- |
|  |  |  | |  | |  |  |  |  |  |
| **localization** | **TCC median** | |  | |  |  | ***MYD88*** | ***CD79B*** | ***CARD11*** | ***BTK*** |
|  |  |  | |  | |  |  |  |  |  |
|  |  |  | |  | |  |  |  |  |  |
| **PMBL** | 50% |  | | mutated cases (%) | |  | 1/12(8) | 1/12(8) | 2/12(16) | 0/12(0) |
|  |  |  | | median AF in % | |  | 11 | 6 | 10 | - |
|  |  |  | |  | |  |  |  |  |  |
| **ENT** | 77% |  | | mutated cases (%) | |  | 13/28(46) | 14/28(50) | 7/28(25) | 1/28(3) |
|  |  |  | | median AF in % | |  | 32 | 36 | 23 | 12 |
|  |  |  | |  | |  |  |  |  |  |
| **CNS** | 70% |  | | mutated cases (%) | |  | 19/26(73) | 15/26(58) | 7/24(29) | 0/24(0) |
|  |  |  | | median AF in % | |  | 36 | 34 | 36 | - |
|  |  |  | |  | |  |  |  |  |  |
| **Testis** | 90% |  | | mutated cases (%) | |  | 4/7(57) | 4/7(57) | 0/7(0) | 0/7(0) |
|  |  |  | | median AF in % | |  | 18 | 29 | - | - |
|  |  |  | |  | |  |  |  |  |  |
| **Breast** | 60% |  | | mutated cases (%) | |  | 3/4(75) | 2/4(50) | 0/4(0) | 1/4(25) |
|  |  |  | | median AF in % | |  | 45 | 68 | - | 43 |
|  |  |  | |  | |  |  |  |  |  |
| **Gastric** | 35% |  | | mutated cases (%) | |  | 2/10(20) | 1/10(10) | 3/10(30) | 1/10(10) |
|  |  |  | | median AF in % | |  | 18 | 6 | 32 | 18 |
|  |  |  | |  | |  |  |  |  |  |
| **Skin** | 60% |  | | mutated cases (%) | |  | 8/16(50) | 6/16(37) | 1/16(6) | 1/16(6) |
|  |  |  | | median AF in % | |  | 25 | 45 | 51 | 51 |
|  |  |  | |  | |  |  |  |  |  |
| **Bone** | 60% |  | | mutated cases (%) | |  | 1/8(12) | 1/8(12) | 0/8(0) | 0/8(0) |
|  |  |  | | median AF in % | |  | 6 | 50 | - | - |
|  |  |  | |  | |  |  |  |  |  |
| **Spleen** | 70% |  | | mutated cases (%) | |  | 0/2(0) | 1/2(50) | 0/2(0) | 0/2(0) |
|  |  |  | | median AF in % | |  | - | 50 | - | - |
|  |  |  | |  | |  |  |  |  |  |
|  |  |  | |  | |  |  |  |  |  |
|  |  |  | |  | |  |  |  |  |  |
|  |  |  | |  | |  |  |  |  |  |
|  |  |  | |  | |  |  |  |  |  |

**Abbreviations**: AF, allele frequency; CNS, central nervous system; ENT, ear nose throat; PMBL, primary mediastinal B-cell lymphoma; TCC, tumour cell content
